# Exploratory Evaluation of the Predictive Value of Serum Neurofilament Light Chain for Autonomic Neuropathy in Hereditary Transthyretin Amyloidosis

**DOI:** 10.3390/jcm15051862

**Published:** 2026-02-28

**Authors:** Milou Berends, Anne Floor Brunger, Hendrea S. A. Tingen, Johan Bijzet, Charlotte E. Teunissen, Paul A. van der Zwaag, Reinold O. B. Gans, Bouke P. C. Hazenberg, Fiete Lange, Gea Drost, Walter Noordzij, Hans L. A. Nienhuis, Riemer H. J. A. Slart

**Affiliations:** 1 Department of Internal Medicine, University Medical Center Groningen, 9700 RB Groningen, The Netherlands; 2 Amyloidosis Center of Expertise, University Medical Center Groningen, 9700 RB Groningen, The Netherlandsb.p.c.hazenberg@umcg.nl (B.P.C.H.);; 3 Department of Rheumatology & Clinical Immunology, Martini Hospital, 9728 NT Groningen, The Netherlands; 4 Department of Nuclear Medicine and Molecular Imaging, University Medical Center Groningen, 9700 RB Groningen, The Netherlands; 5 Department of Laboratory Medicine, University Medical Center Groningen, 9700 RB Groningen, The Netherlands; 6 Neurochemistry Laboratory, Department of Laboratory Medicine, Amsterdam Neuroscience, Amsterdam UMC, Vrije Universiteit Amsterdam, 1081 HV Amsterdam, The Netherlands; 7 Department of Genetics, University Medical Center Groningen, 9700 RB Groningen, The Netherlands; 8 Department of Rheumatology & Clinical Immunology, University Medical Center Groningen, 9700 RB Groningen, The Netherlands; 9 Department of Neurology, University Medical Center Groningen, 9700 RB Groningen, The Netherlands; 10 Biomedical Photonic Imaging Group, Faculty of Science and Technology, University of Twente, 7522 NB Enschede, The Netherlands

**Keywords:** autonomic neuropathy, composite autonomic symptom score 31, Ewing battery, hereditary transthyretin amyloidosis, iodine-123 labeled metaiodobenzylguanidine, serum neurofilament light chain

## Abstract

**Background/Objectives:** Serum neurofilament light chain (sNfL) is a biomarker for peripheral neuropathy as sNfL correlates with polyneuropathy severity in hereditary transthyretin (ATTRv) amyloidosis. It is unclear whether sNfL also correlates with autonomic neuropathy (ANP). In this exploratory study, we aimed to evaluate the value of sNfL as marker for ANP in patients with ATTRv amyloidosis. **Methods:** sNfL was measured retrospectively in 10 pathogenic transthyretin gene variant (*TTR*v) carriers and 28 patients with ATTRv amyloidosis. All 38 individuals underwent a comprehensive evaluation for ANP. **Results:** Individuals with ANP had a higher median sNfL level compared to those without ANP (*p* < 0.001). In univariable logistic regression analysis, age-adjusted sNfL status (normal versus abnormal for age) was associated with sex, ANP, and peripheral neuropathy. In multivariable logistic regression analysis, only peripheral neuropathy significantly predicted age-adjusted sNfL status (normal versus abnormal for age), and no signal was detected for ANP. Receiver operating characteristic analysis showed a considerable area under the curve for ANP. However, the confidence interval was wide for both analyses and only four cases with isolated ANP were included. **Conclusions:** Therefore, in this exploratory cohort, sNfL could not be identified as a marker for ANP, and larger studies are needed to clarify its value.

## 1. Introduction

Hereditary transthyretin (ATTRv) amyloidosis is an autosomal dominant disease with a progressive and fatal course if left untreated. It is caused by pathogenic variants in the gene encoding transthyretin (TTR) [1,2]. Over 150 different pathogenic *TTR* gene variants (*TTR*v) have been described that can cause the TTR tetramer to become unstable, to dissociate into monomers that aggregate into insoluble amyloid fibrils deposited into the extracellular space of tissues and organs [2,3,4]. The most common disease manifestations are autonomic neuropathy (ANP), peripheral neuropathy, and/or cardiomyopathy [4,5,6,7]. Autonomic manifestations, such as orthostasis, vasomotor, gastrointestinal, bladder and pupillomotor dysfunction, often occur in the early stages of ATTRv amyloidosis. The neuropathy in ATTRv amyloidosis is characterized by early involvement of somatic thin myelinated Aδ and autonomic unmyelinated C fibers, resulting in a length-dependent, symmetrical syndrome of loss of temperature sensation, autonomic symptoms, and burning neuropathic pain. As the disease progresses, large myelinated fibers also become impaired [8,9,10].

In the last decade, several disease-modifying treatments have become available for ATTRv amyloidosis. These treatments are most beneficial when initiated at an early disease stage [6,11,12,13]. Therefore, systematic and regular monitoring of asymptomatic *TTR*v carriers to identify the earliest stages of the disease is recommended to improve the outcome.

Neurofilament light chain (NfL) is a neuron-specific cytoskeletal protein released into the blood and cerebrospinal fluid during axonal damage [14]. Recent studies demonstrated that serum neurofilament light chain (sNfL) is released following axonal damage to thick myelinated Aβ nerve fibers, leading to polyneuropathy [15,16]. sNfL serves as a biomarker for polyneuropathy in *TTR*v carriers and ATTRv amyloidosis patients and indicates early disease onset [16]. Axonal damage to the thin myelinated Aδ and unmyelinated C nerve fibers causes ANP [15]. Therefore, sNfL may be a biomarker not only for polyneuropathy but also for ANP. However, there currently is no evidence regarding the role of sNfL as an early marker in ANP [16].

The autonomic nervous system comprises two divisions: the sympathetic and parasympathetic. Several tests are available for assessing autonomic nervous system function. The Ewing battery comprises several autonomic function tests and evaluates both sympathetic and parasympathetic cardiovascular autonomic dysfunction [17,18]. Iodine-123 labeled metaiodobenzylguanidine ([^123^I]*m*IBG) scintigraphy is a nuclear imaging method used to assess the sympathetic conducting system of the heart. [^123^I]*m*IBG is a chemically modified analog of norepinephrine that normally accumulates in vesicles at sympathetic nerve endings near cardiomyocytes. [^123^I]*m*IBG scintigraphy reflects the sympathetic tone, making it useful for evaluating the sympathetic innervation in conditions such as cardiac amyloidosis [19,20,21].

This exploratory study aims to evaluate whether sNfL can serve as a biomarker for ANP in *TTR*v carriers and ATTRv amyloidosis patients. To this end, ANP was evaluated by the Ewing battery, [^123^I]*m*IBG scintigraphy, and autonomic symptom assessment.

## 2. Materials and Methods

### 2.1. Study Participants

**Figure 1:**
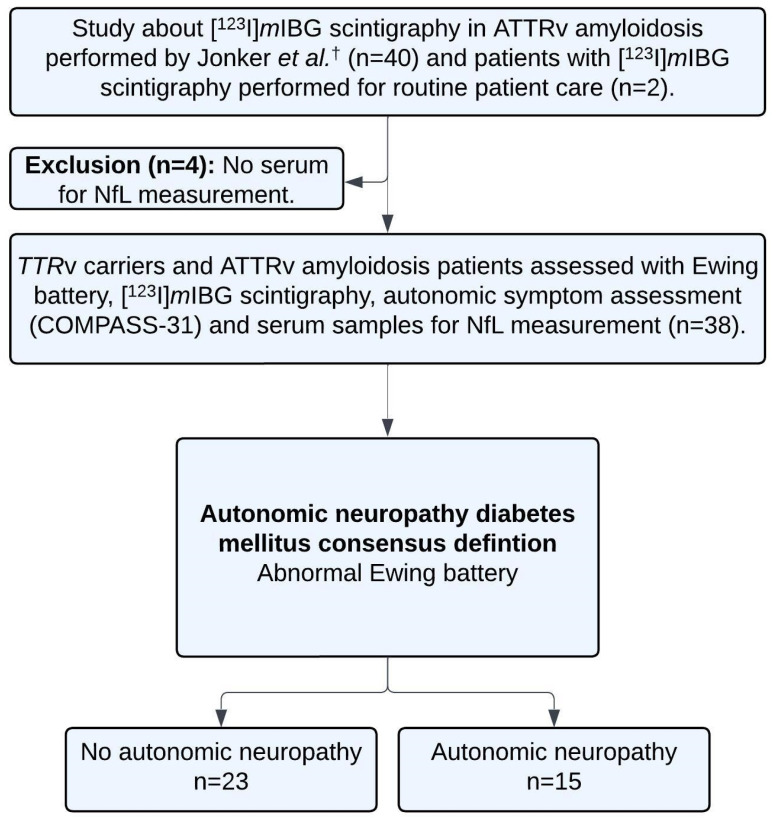
Study inclusion overview. ^†^ Jonker et al.: see reference [22]. ATTRv: hereditary transthyretin amyloid; COMPASS: Composite Autonomic Symptom Score; NfL: neurofilament light chain; *TTR*v: transthyretin gene variant carrier; [^123^I]*m*IBG: iodine-123 labeled metoiodobenzylguanidine.

In a previous study conducted at the University Medical Center Groningen (UMCG) between November 2007 and October 2017, 40 *TTR*v carriers and ATTRv amyloidosis patients underwent a comprehensive assessment [22]. This included Ewing battery, [^123^I]*m*IBG scintigraphy, nerve conduction studies (NCS), quantitative sensory testing (QST), ^99m^Technetium-hydroxymethylene diphosphonate ([^99m^Tc]Tc-HDP) scintigraphy, and subcutaneous fat tissue aspirates. Four patients from the previous study were excluded due to the absence of serum samples for NfL measurement at the time of [^123^I]*m*IBG scintigraphy. Two additional patients were included in the current study cohort having undergone all previously mentioned tests between October 2017 and April 2018 for the purposes of routine patient care (Figure 1).

Serum samples for NfL measurement and subcutaneous fat tissue aspirates within one year before or after [^123^I]*m*IBG scintigraphy were included. Levels of creatinine, N-terminal pro-brain-type natriuretic peptide (NT-proBNP) and troponin T at the time of sNfL measurement, as well as the medical history and results of physical examination, were retrieved from the electronic patient records. For an overview of in- and exclusion criteria, see Table S1.

In this study, *TTR*v carriers were defined as carriers of a pathogenic *TTR*-gene variant without symmetrical distal neuropathic symptoms, or signs of sensory loss and negative NCS and QST; no signs of ANP on Ewing battery and [^123^I]*m*IBG scintigraphy; no signs of cardiomyopathy on [^99m^Tc]Tc-HDP scintigraphy; and no amyloid deposits in the subcutaneous fat tissue aspirate. ATTRv amyloidosis patients were defined as carriers of a pathogenic *TTR*-gene variant with symmetrical distal neuropathic symptoms, or signs of sensory loss that had to be confirmed by NCS or QST, and/or ANP confirmed by Ewing battery and/or [^123^I]*m*IBG scintigraphy, and/or signs of cardiomyopathy on [^99m^Tc]Tc-HDP scintigraphy showing any grade of cardiac tracer uptake (i.e., Perugini grade ≥ 1), and/or a positive subcutaneous fat tissue aspirate (i.e., Congo red score ≥ 1).

### 2.2. Ewing Battery

The Ewing battery was used to detect cardiac ANP and included heart rate variability during deep breathing, heart rate response to Valsalva maneuver, and heart rate response to standing up to assess parasympathetic function, as well as blood pressure response to the isometric handgrip test and standing up to evaluate sympathetic function. Parasympathetic dysfunction was diagnosed if at least two parasympathetic function tests are abnormal, and sympathetic dysfunction was diagnosed if one or both of the sympathetic function tests are abnormal [18,23].

### 2.3. [^123^I]mIBG Scintigraphy

[^123^I]*m*IBG scintigraphy was performed as described [24]. Briefly, after blockage of thyroid uptake by oral administration of iodine potassium iodide, patients were intravenously injected with 185 MBq [^123^I]*m*IBG. Planar images of the thorax were acquired at fifteen minutes and at four hours after radiotracer injection to quantify the early and late heart-to-mediastinum ratio (HMR) [24]. HMR was determined by the counts in a manually drawn region of interest (ROI) along the contour of the left ventricle, divided by the counts in a fixed rectangular ROI in the upper mediastinum (carefully leaving the thyroid region out). The cardiac wash-out rate (WR) was defined as a change in percentage of the activity ratio, calculated as follows: $\frac{{HMR}_{early}-\;{HMR}_{late}}{{HMR}_{early}}*100\%$ [19,21,24,25,26]. Either late HMR < 2.0 or WR > 20% were considered as abnormal [^123^I]*m*IBG parameters, and hence suggestive of impaired sympathetic cardiac innervation [27,28,29].

### 2.4. Patient-Reported Autonomic Symptoms

Symptoms as described in the Composite Autonomic Symptom Score (COMPASS) 31 were used to assess the presence of ANP symptoms [30].

### 2.5. Autonomic Neuropathy

According to expert panel consensus in diabetes mellitus patients, the Ewing battery is the gold standard for the assessment of ANP. Parasympathetic dysfunction is diagnosed if at least two parasympathetic function tests are abnormal, and sympathetic dysfunction is diagnosed if one or both of the sympathetic function tests are abnormal. We chose not to subcategorize autonomic dysfunction into sympathetic and parasympathetic components to maintain maximum statistical power for analysis. Therefore, Ewing battery results were reported as either normal or abnormal (*ANP consensus definition*) [18,23]. As there is no validated consensus definition for ANP in ATTRv amyloidosis patients, we used a modified definition for ANP (*ANP modified definition*), which requires abnormal results in at least two of the following three assessments: Ewing battery, [^123^I]*m*IBG scintigraphy, and/or the presence of autonomic symptoms as assessed by the COMPASS-31 questionnaire [30]. Both definitions were applied to the same patient population. As patient classification was nearly identical regardless of the definition applied, only the diabetes consensus definition, based solely on the Ewing battery, was used for data analysis.

### 2.6. Peripheral Neuropathy

QST was performed to detect small fiber neuropathy (SFN) [13,31]. NCS were used to detect large fiber neuropathy [13,32]. Peripheral neuropathy, including both small and large fiber neuropathy, was defined as symmetrical distal neuropathic symptoms, or signs of sensory loss and had to be confirmed by NCS or QST. The severity of the peripheral neuropathy was graded according to the polyneuropathy disability (PND) score. The PND score indicates how walking is affected: 0: No disturbance; I: Sensory disturbance; II: Walking difficulties, not requiring support or care; IIIa: Need of one stick or one crutch for ambulation; IIIb: Need of two sticks or two crutches for ambulation; IV: Patient confined to a bed or wheelchair [33].

### 2.7. Blood Sample Collection and NfL Measurement

Blood samples were drawn by venipuncture at the outpatient clinic of the UMCG, centrifuged at 2700 rpm for 10 min at room temperature and were stored within one hour at −20 °C and subsequently stored at −80 °C within six months. Measurements of NfL in serum were performed using the NF-light Kit (Quanterix) on a single molecule array (Simoa HDX) technology (Quanterix Corp., Billerica, MA, USA) [34]. The measurements were performed at the Neurochemistry Laboratory of the Amsterdam University Medical Center by certified technicians who were blinded to clinical information.

### 2.8. Statistical Analysis

The Kolmogorov–Smirnoff test was used to assess the normality of the data. Patient characteristics were described as number (*n*), mean ± standard deviation (SD) when normally distributed, and median [interquartile range (IQR)] when not normally distributed. Groups were compared using the unpaired t-test, Mann–Whitney U test or Chi-square test (for trend), where appropriate. Univariable correlations were assessed by Spearman’s rho correlation coefficient.

Logistic regression analysis was performed to assess whether sNfL levels predicted ANP. sNfL levels were first converted into a dichotomous variable (normal versus abnormal for age), based on normative data from Vermunt et al. [35]. In univariable logistic regression, all relevant clinical variables were tested individually with enter inclusion of the variables. Variables that were significant in univariable logistic regression analysis were included in the multivariable logistic regression model using enter inclusion. The diagnostic performance of sNfL for the presence of neuropathy was assessed using receiver operating characteristic (ROC) analysis. The area under the curve (AUC) was calculated to quantify the discriminative ability of sNfL in predicting the presence of neuropathy. A *p*-value < 0.05 was considered statistically significant.

IBM SPSS Statistics version 28.0 (IBM Corp., Armonk, NY, USA) was used for statistical analysis. GraphPad Prism 9 (GraphPad Software, La Jolla, CA, USA) was used to generate graphs.

### 2.9. Ethics Approval

All procedures were in compliance with the Declaration of Helsinki. The study was approved by the institutional review board of the UMCG (Registration number 201800204 and 201900860). According to the local Dutch regulations for retrospective observational studies, formal informed consent was not required.

## 3. Results

### 3.1. Characteristics of TTRv Carriers and ATTRv Amyloidosis Patients

A total of 38 individuals (19 male and 19 female) were included, of which 10 were *TTR*v carriers and 28 ATTRv amyloidosis patients. The overall mean age at the time of sNfL measurement was 50 ± 14 years. Most frequent (45%) was the Val30Met (p.Val50Met) genotype. See Table S2 for a specification of genotypes. Peripheral neuropathy was diagnosed in 17 individuals. Twenty individuals had PND score 0, including nine *TTR*v carriers and 11 ATTRv amyloidosis patients without peripheral neuropathy but with autonomic neuropathy, cardiomyopathy, and/or confirmed amyloid deposits on fat tissue biopsy. Cardiomyopathy was diagnosed in 14 individuals. Autonomic symptoms listed on the COMPASS-31 questionnaire were reported by 14 individuals [30], abnormal results on the Ewing battery by 15 individuals, and abnormal findings on [^123^I]*m*IBG scintigraphy by 15 individuals. There was a negative correlation between sNfL and late HMR (r = −0.62, *p* < 0.001) and a positive correlation between sNfL and WR (r = 0.64, *p* < 0.001). ANP was diagnosed in 15 individuals. A total of 16 individuals received treatment, either with diflunisal (*n* = 10) or tafamidis (*n* = 6) at the time of blood collection (Table 1).

### 3.2. sNfL in Relation to Autonomic Neuropathy

**Figure 2:**
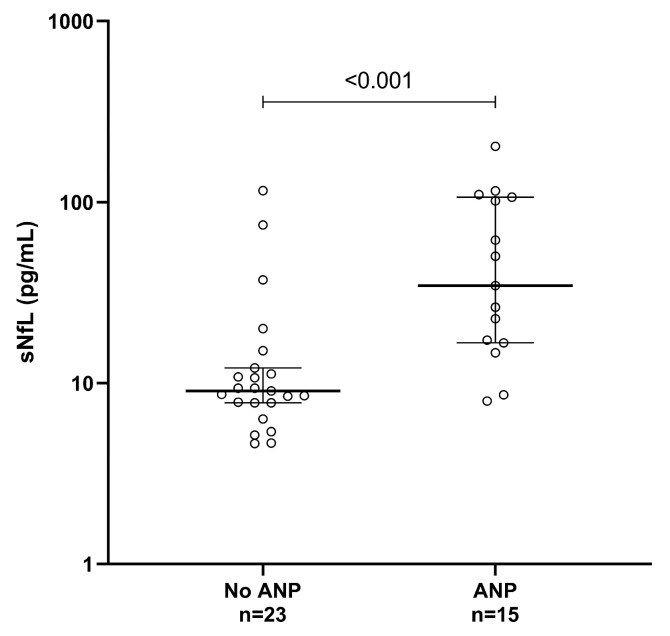
sNfL in relation to autonomic neuropathy. sNfL levels are displayed as median [interquartile range]. A *p*-value < 0.05 was deemed significant. ANP: autonomic neuropathy; sNfL: serum neurofilament light chain.

Thirteen pathogenic *TTR*v carriers had an abnormal age-adjusted sNfL status (Table S3) [35]. The median sNfL level was higher in individuals with ANP (*n* = 15) compared to those without ANP (*n* = 23), 34.6 pg/mL [16.7–106.5] and 9.1 pg/mL [7.8–12.1] (*p* < 0.001), respectively (Table 2 and Figure 2).

Table 2 presents the median sNfL levels based on results from the Ewing battery, [^123^I]*m*IBG scintigraphy, and assessment of autonomic symptoms.

### 3.3. sNfL Levels in Patients with Autonomic Neuropathy and No Peripheral Neuropathy

Median sNfL levels in individuals with only ANP (*n* = 4) did not significantly differ compared to those without any form of neuropathy (*n* = 17) (Figure S1).

### 3.4. Univariable Logistic Regression Analysis

Univariable logistic regression analysis showed that peripheral neuropathy had the highest explained variance for age-adjusted sNfL status based on the Nagelkerke R^2^ (Table 3). The variables sex, autonomic symptoms, Ewing battery, [^123^I]*m*IBG scintigraphy, ANP, and peripheral neuropathy were all significantly associated with age-adjusted sNfL status (*p* < 0.05) (Table 3).

### 3.5. Multivariable Logistic Regression Analysis

In the multivariable logistic regression model, variables that were significant in univariable logistic regression were included. The dependent variable age-adjusted sNfL status and the independent variables sex, ANP, and peripheral neuropathy were included (Table 4). Among these, only peripheral neuropathy (odds ratio (OR) 25.03 95% confidence interval (CI) 2.33–269.26) was identified as independent predictor for age-adjusted sNfL status (*p* < 0.05). The Nagelkerke R^2^ for peripheral neuropathy improved from 0.57 in univariable logistic regression analysis to 0.64 in this model.

### 3.6. ROC Analysis

ROC analysis of sNfL was performed to assess its diagnostic performance for detecting any neuropathy, peripheral neuropathy, and ANP (Figure S2A–C). For predicting any neuropathy, sNfL showed good discriminative ability, with an of 0.87 (95% CI 0.75–0.99). The AUC for sNfL in predicting peripheral neuropathy was 0.91 (95% CI 0.79–1.00), and 0.92 (95% CI 0.82–1.00) for ANP.

## 4. Discussion

This study shows that (1) sNfL levels are increased in ATTRv amyloidosis patients with ANP (2); however, multivariate logistic regression analysis did not identify an independent association with ANP. ROC analysis showed a substantial AUC for ANP, but this is likely driven by the concomitant presence of polyneuropathy in the majority of patients. Therefore, based on the results of this exploratory cohort, sNfL could not be identified as a biomarker to detect ANP in pathogenic *TTR*v carriers and ATTRv amyloidosis patients. This emphasizes the continued necessity of current screening and diagnostic methods for the detection of ANP.

NfL is released into the blood following peripheral neuronal damage, particularly to thick myelinated Aβ nerve fibers [15,16]. Axonal damage to the somatic thin myelinated Aδ and autonomic unmyelinated C nerve fibers may also result in the release of NfL [15]. Therefore, we hypothesized that NfL could serve as a biomarker for ANP.

In line with our hypothesis, we observed increased sNfL levels in individuals with ANP compared to those without ANP (Figure 2). To further investigate the relationship between sNfL and ANP, we performed logistic regression analysis to assess whether ANP could be predicted based on age-adjusted sNfL status. In multivariable logistic regression analysis, only peripheral neuropathy significantly predicted age-adjusted sNfL status (normal versus abnormal for age), and no signal was detected for ANP. Individuals with peripheral neuropathy had approximately 25 times higher odds of having an abnormal age-adjusted sNfL status compared to those without peripheral neuropathy (Table 4). The multivariable regression analysis should be interpreted with caution, as the high OR with wide CI indicates uncertainty. Consequently, residual confounding between peripheral neuropathy and ANP cannot be excluded.

In line with the results of logistic regression analysis, we observed that the median sNfL levels did not significantly differ between individuals with only ANP (*n* = 4) compared to those individuals without any form of neuropathy (*n* = 17) (Figure S1). Furthermore, ROC analysis indicated that sNfL has good discriminative ability for detecting any neuropathy, peripheral neuropathy and ANP. However, the ROC analysis for ANP was largely driven by peripheral neuropathy, as the majority of patients with ANP also had peripheral neuropathy. Only four patients had isolated ANP. This small number of patients also did not allow a meaningful ROC analysis for isolated autonomic neuropathy. Consequently, no definitive conclusions can be drawn from this exploratory study regarding the value of sNfL as biomarker for ANP. ANP may contribute to sNfL levels. However, if ANP does contribute to sNfL levels, its contribution is probably rather small compared with the contribution of peripheral neuropathy.

Few studies have investigated the role of sNfL in ANP, limiting direct comparisons. Luigetti et al. investigated correlations between sNfL and systemic disease parameters in patients with ATTRv amyloidosis [36]. They used the questionnaire-based compound autonomic dysfunction test (CADT) and Sudoscan to assess autonomic dysfunction. Significant correlations were reported between NfL levels and Sudoscan parameters, but not with CADT scores. However, they did not correct for the presence of polyneuropathy [36]. Similarly, Russo et al. observed a significant correlation between sNfL and disease severity in ATTRv amyloidosis patients, including autonomic dysfunction as assessed by the CADT [37]. However, this study also lacked adjustment for large fiber neuropathy. Another study in patients with type II diabetes mellitus with or without diabetic neuropathy assessed heart rate variability measures and sNfL levels [38]. After adjusting for glycemic control, they found significant correlations between sNfL and the heart rate variability. This suggests that sNfL could play a role in identifying individuals at risk of cardiac ANP. However, as with the aforementioned studies, they did not adjust for the presence of peripheral neuropathy [38].

Taken together, our findings and those from the aforementioned studies indicate a potential association between sNfL and autonomic dysfunction [36,37,38]. However, the impact of confounding factors, particularly co-existing peripheral neuropathy, has not been thoroughly investigated. As ANP is a form of SFN that affects the thin myelinated Aδ and unmyelinated C nerve fibers, we reviewed the literature on SFN and NfL [15]. Galosi et al. assessed sNfL levels and SFN in ATTRv amyloidosis [39]. Consistent with previous studies [40,41,42], they found that intraepidermal nerve fiber density (IENFD) and thermal thresholds measured by QST are often impaired early, even in asymptomatic *TTR*v carriers. Furthermore, they observed a significant negative correlation between sNfL and distal IENFD, and sNfL levels significantly correlated with QST measures indicating small fiber impairment. However, they did not account for the presence of polyneuropathy in their analysis, nor did they compare sNfL levels between asymptomatic *TTR*v carriers with normal versus abnormal IENFD and QST results [39]. In another study, sNfL levels were investigated in a cohort of patients with SFN of various etiologies and they showed that sNfL levels did not correlate with any parameters of small nerve fiber function [43]. Both studies suggested that either the NfL content of small intraepidermal nerve fibers is too low, or the axonal damage in SFN is generally too subtle to detect altered sNfL levels [39,43].

In our cohort, four of the 38 pathogenic *TTR*v carriers (10%) presented with isolated ANP, and 11 of the 38 pathogenic *TTR*v carriers (29%) had peripheral neuropathy as well as ANP. The incidence of isolated ANP in ATTRv amyloidosis is not well described in the literature. Data from the Transthyretin Amyloidosis Outcomes Survey showed that ANP was the first presenting symptom category in 37.3% of Val30Met (p.Val50Met) and 12.5% of non-Val30Met (non-p.Val50Met) patients [44]. However, it was not reported whether these cases involved ANP exclusively. As autonomic symptoms can be among the earliest disease manifestations, it is important to screen for ANP. Previous studies in small patient groups have shown that sNfL can be useful in detecting neuropathy even in a presymptomatic stage [45,46]. To date, no other biomarkers have been identified for ANP in ATTRv amyloidosis. However, glial fibrillary acidic protein (GFAP), expressed in the central, peripheral, and enteric nervous system, may serve as a potential biomarker for the detection of ANP [47]. A study by Plantone et al. found that serum GFAP levels were elevated in both asymptomatic *TTR*v carriers and symptomatic ATTRv amyloidosis patients, reflecting early disease activity [48]. As GFAP is expressed not only in the central nervous system astrocytes but also in non-myelinating Schwann cells and enteric glial cells [47], its elevation may originate from subclinical peripheral or enteric nervous system involvement. Therefore, GFAP may represent a promising biomarker for peripheral SFN and ANP in ATTRv amyloidosis. However, until such data become available, current diagnostic methods remain necessary for the evaluation of ANP.

### 4.1. Limitations

Clear limitations of this exploratory study are its retrospective design and the small number of *TTR*v carriers and ATTRv amyloidosis patients with isolated ANP that could be included, which limits the power to draw definitive conclusions.

We recognize that dichotomizing sNfL reduces the amount of information and statistical power. In addition, we performed multiple statistical tests on a relatively small sample size. Although our study aimed to explore potential patterns rather than establish confirmatory evidence, we acknowledge that conducting multiple tests increases the risk of inflated type I error. Therefore, we emphasize that these findings should be interpreted as exploratory and hypothesis-generating rather than definitive.

Treatment with TTR stabilizers and renal function could theoretically affect sNfL levels. However, neither treatment status nor creatinine levels were associated with sNfL in univariate analyses. Given the limited sample size of our cohort, we therefore did not include these variables in the multivariate model, as doing so would have increased the risk of overfitting. Nonetheless, one should acknowledge the possibility of residual confounding related to treatment and renal function.

Furthermore, this study was limited to ANP. Although an assessment of SFN in relation to sNfL would also have been valuable, such analysis was not feasible due to insufficient data from QST. Furthermore, IENFD assessment via skin biopsy is not routinely performed at our center.

Lastly, ANP was defined based on the results of the Ewing battery, [^123^I]*m*IBG scintigraphy, and COMPASS-31 questionnaire. The Ewing battery and [^123^I]*m*IBG scintigraphy primarily assess cardiovascular ANP, whereas the COMPASS-31 provides a broader evaluation of autonomic function. Consequently, cardiovascular ANP is mainly reflected in our study. Including additional objective tests for non-cardiovascular autonomic function (e.g., Sudoscan, and quantitative sudomotor axon reflex test (QSART)) would have provided a more comprehensive assessment of ANP. Unfortunately, too few Sudoscan data and no QSART data were available for analysis.

### 4.2. Further Considerations

Although sNfL has shown to be a useful biomarker for the early detection and monitoring of peripheral neuropathy in ATTRv amyloidosis [16], this exploratory study and other studies suggest that sNfL is not sensitive enough to detect peripheral SFN and ANP [39,43]. Consequently, in clinical practice, ANP should still be assessed by tools such as the Ewing battery, [^123^I]*m*IBG scintigraphy, Sudoscan, and/or the COMPASS-31 questionnaire. Of these, [^123^I]*m*IBG scintigraphy is particularly promising for the early detection of cardiac ANP [22]. It has shown to be more sensitive than bone scintigraphy, with the ability to detect cardiac sympathetic denervation prior to the emergence of structural heart abnormalities, clinical symptoms, or positive findings on other imaging modalities [22,25,49,50]. For assessing sudomotor function, the measurement of feet electrochemical skin conductance using the Sudoscan has shown to be a sensitive and non-invasive method for detecting early autonomic dysfunction in ATTRv amyloidosis patients [40,51].

For peripheral SFN, QST and skin biopsies to assess IENFD remain essential. Notably, IENFD has found to be positive in asymptomatic *TTR*v carriers, which highlights its importance in the early detection of ATTRv amyloidosis [39,40].

## 5. Conclusions

This exploratory study demonstrates that sNfL levels are increased in ATTRv amyloidosis patients with ANP, but do not show an independent association with ANP after adjustment for co-existing peripheral neuropathy. Given the retrospective design, the small overall sample size, and especially the limited number of patients with isolated ANP, this study lacks sufficient power to draw definitive conclusions. Current evidence supports sNfL as a suitable biomarker for the detection and monitoring of large fiber neuropathy. However, based on the available data, no firm conclusions can be drawn regarding its ability to reliably detect ANP or SFN. This is particularly relevant in the context of using sNfL as an early biomarker for ATTRv amyloidosis, as ANP and SFN may represent the first clinical manifestations in a subset of patients. Larger, prospective, multicenter studies, including a broad range of genotypes and preferably focusing on *TTR*v carriers transitioning from asymptomatic to symptomatic disease, are needed to definitively establish the value of sNfL as an early biomarker for ATTRv amyloidosis. Until such data become available, comprehensive clinical assessment remains essential.

## Figures and Tables

**Table 1:** Patient characteristics.

			Total (*n* = 38)
**General**			
	Gender (male/female)		19/19
	Age ± SD (years)		50 ± 14
	Val30Met (p.Val50Met)/non-Val30Met (non p.Val50Met)		17/21
	Amyloid grade of the Congo red-stained fat tissue aspirate		
		No amyloid	15
		1+	6
		2+	5
		3+	8
		4+	4
	Treatment		
		Diflunisal	10
		Tafamidis	6
	Creatinine (µmol/L)		76 ± 15
**Autonomic neuropathy (absent/present)**			23/15
	Ewing battery abnormal (no/yes)		23/15
	[^123^I]*m*IBG scintigraphy abnormal (no/yes)		23/15
	Late heart-to-mediastinum ratio		2.3 ± 0.8
	Wash-out rate (%)		5.1 ± 14.7
	Autonomic symptoms ^§^		
		Cardiac (no/yes)	30/8
		Gastro-intestinal (no/yes)	30/8
		Urogenital (no/yes)	31/7
		Visual (no/yes)	38/0
**Peripheral neuropathy ^¶^ (absent/present)**			21/17
	NCS (normal/abnormal)		21/17
	QST (normal/abnormal)		14/24
	Signs and symptoms of sensory loss (no/yes)		20/18
	PND score		
		0	20
		I	16
		>I	2
**Cardiomyopathy (absent/present)**			24/14
	NT-proBNP (ng/L)		78 [37–277]
	Troponin T (ng/L)		5 [2–13]
	[^99m^Tc]Tc-HDP scintigraphy (normal/abnormal)		24/14

^§^ Autonomic symptoms: symptoms as described in the COMPASS-31 questionnaire [30]. ^¶^ Peripheral neuropathy definition: symmetrical distal neuropathic symptoms, or signs of sensory loss confirmed by NCS/QST. COMPASS: Composite Autonomic Symptom Score; NCS: nerve conduction studies; NT-proBNP: N-terminal pro-brain-type natriuretic peptide; PND: polyneuropathy disability; QST: quantitative sensory testing; sNfL: serum neurofilament light chain; [^123^I]*m*IBG: iodine-123 labeled metaiodobenzylguanidine; [^99m^Tc]Tc-HDP scintigraphy: ^99m^Technetium-hydroxymethylene diphosphonate.

**Table 2:** sNfL levels.

	sNfL (pg/mL)	sNfL (pg/mL)	*p*-Value
	**No autonomic neuropathy**	**Autonomic neuropathy**	
Ewing battery	9.1 [7.8–12.1] *n* = 23	34.6 [16.7–106.5] *n* = 15	<0.001
	**Normal/absent**	**Abnormal/present**	
[^123^I]*m*IBG scintigraphy	9.1 [7.8–14.7] *n* = 23	61.8 [16.7–110.1] *n* = 15	<0.001
Autonomic symptoms ^§^	9.2 [7.8–13.6] *n* = 24	43.7 [16.7–106.5] *n* = 14	0.002

^§^ Autonomic symptoms: symptoms as described in the COMPASS-31 questionnaire [30]. sNfL levels are described as median [interquartile range]. COMPASS: Composite Autonomic Symptom Score; sNfL: serum neurofilament light chain; [^123^I]*m*IBG-scintigraphy: iodine-123 labeled metaiodobenzylguanidine.

**Table 3:** Univariable logistic regression analysis for age-adjusted sNfL status (normal versus abnormal for age).

	OR (95% CI)	Nagelkerke R^2^	*p*-Value
**General**			
Sex (male ^†^/female)	0.09 (0.02–0.48)	0.33	0.01
Creatinine (µmol/L)	1.01 (0.97–1.06)	0.01	0.57
Treatment (no/yes)	2.07 (0.53–8.1)	0.04	0.29
**Autonomic neuropathy**			
Ewing battery abnormal (no ^†^/yes)	7.13 (1.60–31.72)	0.24	0.01
Autonomic symptoms ^‡^ (no ^†^/yes)	9.00 (1.95–41.65)	0.29	0.01
[^123^I]*m*IBG-scintigraphy abnormal (no ^†^/yes)	7.13 (1.60–31.72)	0.24	0.01
**Peripheral neuropathy**			
Peripheral neuropathy (no ^†^/yes)	48.00 (4.99–461.45)	0.57	<0.001

Results of univariable logistic regression analysis with age-adjusted sNfL status (normal versus abnormal for age) as the dependent variable. Enter inclusion of variables was performed. Reference categories are indicated with ^†^. ^‡^ Autonomic symptoms: symptoms as described in the COMPASS-31 questionnaire [30]. CI: confidence interval; COMPASS: Composite Autonomic Symptom Score; OR: odds ratio; sNfL: serum neurofilament light chain; [^123^I]*m*IBG: iodine-123 labeled metaiodobenzylguanidine.

**Table 4:** Multivariable logistic regression analysis for age-adjusted sNfL status (normal versus abnormal for age).

	OR (95% CI)	*p*-Value
Autonomic neuropathy (no ^‡^/yes)	2.26 (0.30–17.01)	0.43
Peripheral neuropathy (no ^‡^/yes)	25.03 (2.33–269.26)	0.01
Sex (male ^‡^/female)	0.21 (0.02–1.75)	0.15

Results of multivariable logistic regression analysis with age-adjusted sNfL status (normal vs. abnormal for age) as the dependent variable. Enter inclusion of variables was performed. Reference categories are indicated with **^‡^**. Nagelkerke R^2^: 0.64. CI: confidence interval; OR: odds ratio; sNfL: serum neurofilament light chain.

## Data Availability

The datasets generated during and/or analyzed during the current study are available from the corresponding author on reasonable request.

## Supplementary Materials

The following supporting information can be downloaded at https://www.mdpi.com/article/10.3390/jcm15051862/s1, Table S1: In- and exclusion criteria; Table S2: Genotype distribution; Table S3: sNfL levels and age-adjusted references values; Figure S1: sNfL levels in patients with autonomic neuropathy compared to those without any form of neuropathy; Figure S2: ROC analysis of sNfL.

## Abbreviations

ANP	autonomic neuropathy
ATTRv	hereditary transthyretin amyloid
AUC	area under the curve
CADT	compound autonomic dysfunction test
CI	confidence interval
COMPASS	Composite Autonomic Symptom Score
GFAP	glial fibrillary acidic protein
HMR	heart-to-mediastinum ratio
IENFD	intraepidermal nerve fiber density
IQR	interquartile range
NCS	nerve conduction studies
NfL	neurofilament light chain
NT-proBNP	N-terminal pro-brain-type natriuretic peptide
OR	odds ratio
PND	polyneuropathy disability
Simoa	single molecule array
sNfL	serum neurofilament light chain
SD	standard deviation
TTR	transthyretin
TTRv	transthyretin gene variant
QST	quantitative sensory testing
ROC	receiver operating characteristics
ROI	region of interest
UMCG	University Medical Center Groningen
WR	wash-out rate
[^123^I]mIBG	iodine-123 labeled metaiodobenzylguanidine
[^99m^Tc]Tc-HDP	99mTechnetium-hydroxymethylene diphosphonate
