# Supplementary material for: Exploratory Evaluation of the Predictive Value of Serum Neurofilament Light Chain for Autonomic Neuropathy in Hereditary Transthyretin Amyloidosis
Source: J Clin Med. 2026 Feb 28;15(5):1862. doi: 10.3390/jcm15051862 (PMC12986165; doi:10.3390/jcm15051862)
Supplement: Supplementary file 1 [file jcm-15-01862-s001.zip › jcm-4141433-supplementary.pdf]

Supplementary Tables

Table S1 In- and exclusion criteria.

	**Inclusion**	**Exclusion**
Study population	Pathogenic *TTR*v carriers or ATTRv amyloidosis patients.	No pathogenic *TTR*v carriers or ATTRv amyloidosis patients.
Center	UMCG	Other centers
Investigations/ retrieval from electronic patient records	- Ewing battery<br>- Iodine-123 labelled metaiodobenzylguanidine scintigraphy<br>- Nerve conduction studies<br>- Quantitative sensory testing<br>- ^{99m}Technetium-hydroxymethylene diphosphonate scintigraphy<br>- Subcutaneous fat tissue aspirates<br>- sNfL<br>- Creatinine<br>- N-terminal pro-brain-type natriuretic peptide<br>- Troponin T	Not all investigations mentioned by inclusions criteria available.
Timing	- sNfL and subcutaneous fat tissue aspirates one year before or after iodine-123 labelled metaiodobenzylguanidine scintigraphy.	- sNfL and subcutaneous fat tissue aspirates more than one year before or after iodine-

	- Creatinine, N-terminal pro-brain-type natriuretic peptide, and troponin T at time of sNfL measurement.	123 labelled metaiodobenzylguanidine scintigraphy.<br>- Creatinine, N-terminal pro-brain-type natriuretic peptide, and troponin T not measured at time of sNfL measurement.

ATTRv: hereditary transthyretin amyloid; *TTR*v: transthyretin variant; sNfL: serum neurofilament light chain; UMCG: university medical center Groningen.

Table S2 Genotype distribution.

TTR-gene variant	Total n (%)
Val30Met (p.Val50Met)	17 (45)
Tyr114Cys (p.Tyr134Cys)	7 (18)
Gly89Lys (p.Glu109Lys)	4 (11)
Val71Ala (p.Val91Ala)	3 (9)
Ser23Asn (p.Ser43Asn)	2 (5)
Val94Ala (p.Val114Ala)	1 (3)
Gly47Glu (p.Gly67Glu)	1 (3)
Ala45Gly (p.Ala65Gly)	1 (3)
Val122del (p.Val142del)	1 (3)
Val122Ile (p.Val142Ile)	1 (3)

TTR: transthyretin

Table S3 sNfL levels and age-adjusted references values.

Pathogenic *TTR*v carrier			sNfL reference value for age 5–95th percentile (pg/mL)
sNfL (pg/mL)	Age (years)	Normal/abnormal sNfL level for age	
4,6	26	normal	3–9
4,7	48	normal	4–14
5,2	43	normal	3–13
5,4	28	normal	3–10
6,3	38	normal	3–12
7,8	39	normal	3–12
7,8	35	normal	3–11
7,8	61	normal	5–19
8,0	54	normal	4–16
8,5	55	normal	4–16
8,5	62	normal	5–19
8,6	46	normal	3–13
8,7	35	normal	3–11
9,1	55	normal	4–16
9,4	42	normal	3–12
9,4	38	normal	3–12
10,7	50	normal	3–14
10,8	37	normal	3–11
11,3	52	normal	4–14
12,1	29	abnormal	3–10
14,7	62	normal	5–19
15,1	63	normal	6–23
16,7	68	normal	7–27
17,3	69	normal	7–27
20,0	64	normal	6–23
22,7	66	normal	6–23
26,3	44	abnormal	3–13
34,6	74	abnormal	9–32
37,2	55	abnormal	4–16
50,2	34	abnormal	3–11
61,8	45	abnormal	3–13
74,7	42	abnormal	3–12
101,8	36	abnormal	3–11

106,5	60	abnormal	5–19
110,1	42	abnormal	3–12
115,3	74	abnormal	9–32
115,7	78	abnormal	10–37
203,7	36	abnormal	3–11

sNfL: serum neurofilament light chain; *TTR*v: transthyretin variant.

Supplementary Figures

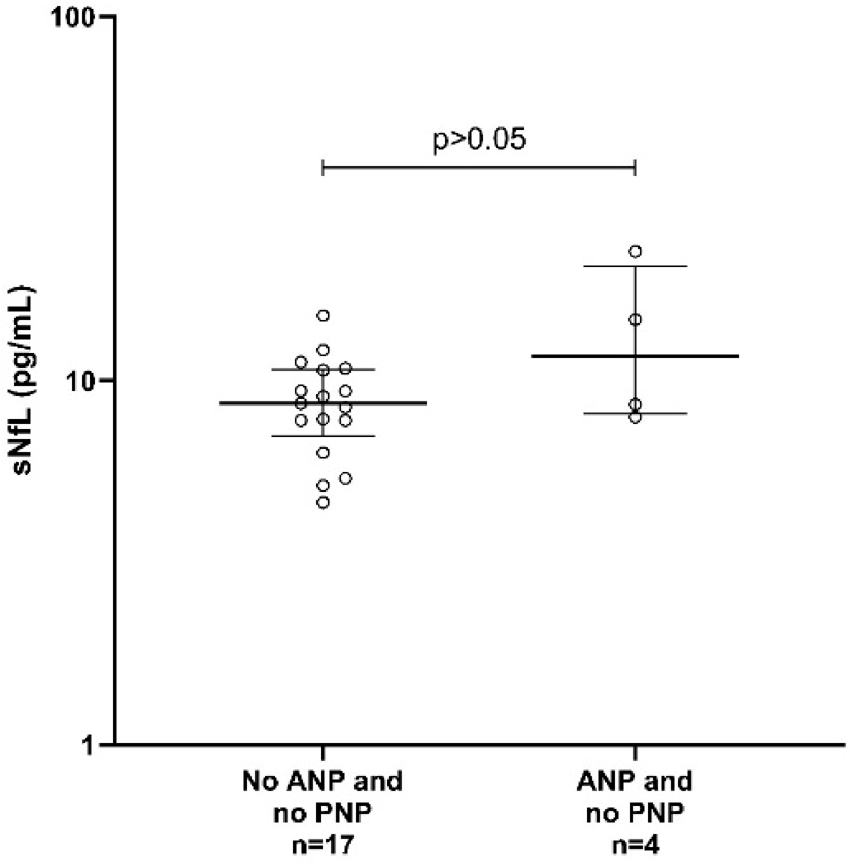

Figure S1. sNfL levels in patients with autonomic neuropathy compared to those without any form of neuropathy.

sNfL levels are displayed as median [interquartile range]. A p-value <0.05 was deemed significant.

ANP: autonomic neuropathy; PNP: peripheral neuropathy; sNfL: serum neurofilament light chain.

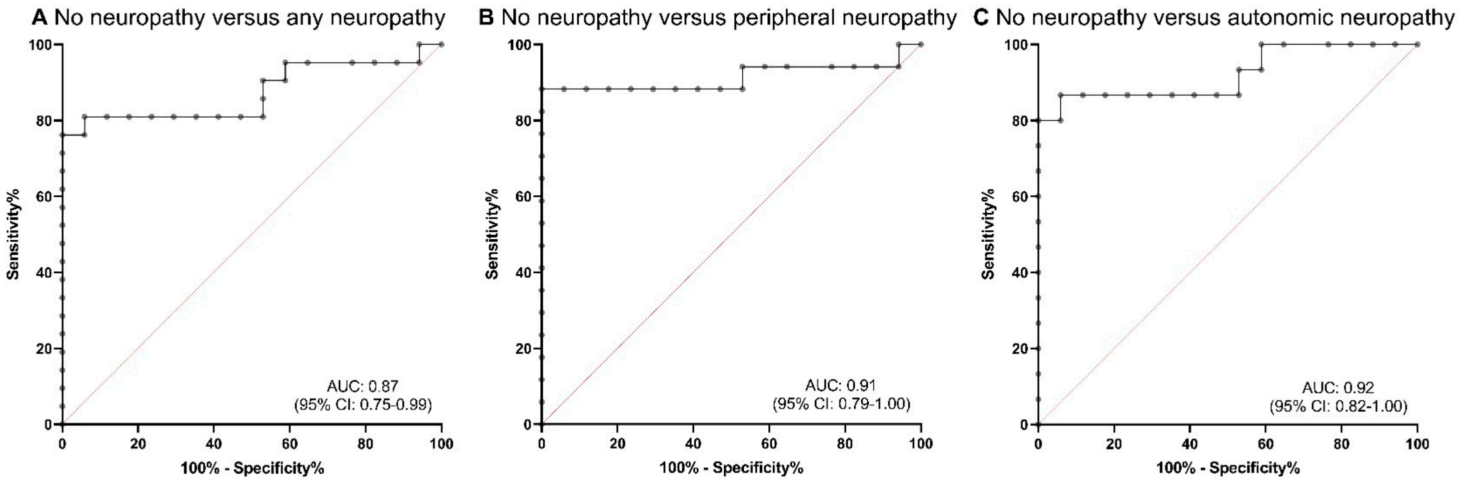

Figure S2. ROC analysis of sNfL.

A No neuropathy (*n* = 17) versus any neuropathy (*n* = 21); **B** No neuropathy (*n* = 17) versus peripheral neuropathy (*n* = 17); **C** No neuropathy (*n* = 17) versus autonomic neuropathy (*n* = 15).

AUC: area under the curve; CI: confidence interval; ROC: receiver operating characteristic; sNfL: serum neurofilament light chain.
